# Supplementary material for: Coevolutionary transitions from antagonism to mutualism explained by the Co-Opted Antagonist Hypothesis
Source: Nat Commun. 2021 May 17;12:2867. doi: 10.1038/s41467-021-23177-x (PMC8129128; doi:10.1038/s41467-021-23177-x)
Supplement: Supplementary file 1 — Supplementary Information [file 41467_2021_23177_MOESM1_ESM.pdf]

SUPPLEMENTARY INFORMATION
Manuscript: "Coevolutionary transitions from antagonism to mutualism explained by the Co-Opted Antagonist Hypothesis"

Supplementary Data 1

Experiment methodology and key empirical results

We performed preference assays to assess *Manduca sexta* floral visitation and oviposition preferences. *M. sexta* individuals from the University of Arizona laboratory colony were sequentially released into a large (22m x 7.5m x 3m) greenhouse containing two potted *D. wrightii* and two potted *D. discolor* plants arranged in a checkered array with ~2m between plants. An evaporative cooling system maintained a temperature of ~26°C and generated directional airflow from the plant array to the release point ~10m downwind. A single moth was released at a time and each floral visit and oviposit was counted. After each trial, the number of eggs laid on each plant were tallied, but not removed. At the end of each night, all eggs were removed from the plants and the flowers were tagged. Plants were then moved to a separate greenhouse and allowed to set seed. Thus, we quantified seed set as a function of the number of moths that visited each flower and the number of eggs that each female moth laid on each plant as a function of the number of floral visits that the moth made to that plant. See [1] for more details.

Moth-visited flowers set significantly more seeds than did autonomously self-pollinated flowers for both *Datura* species (ANOVA: *D. wrightii*, $n = 21$ fruit, $SS = 111057$, $F = 56.6$, $p < 0.001$; *D. discolor*, $n = 85$ fruit, $SS = 185244$, $F = 139$, $p < 0.001$). This result corroborates a previous study on *D. wrightii*[2], but had not been demonstrated for *D. discolor*. Seed set of moth-pollinated flowers was significantly higher for *D. wrightii* than for *D. discolor* (ANOVA: $n = 35$ fruit, $SS = 15560$, $F = 6.46$, $p = 0.02$). There was a trend towards higher seed set in moth cross-pollinated flowers (flowers visited by moths that had previously visited conspecific flowers) than moth self-pollinated flowers (flowers visited by moths that had not previously visited conspecific flowers), but this difference was nonsignificant (ANOVA: *D. wrightii*, $n = 13$ fruit, $SS = 3677$, $F = 1.3$, $p = 0.28$; *D. discolor*, $n = 22$ fruit, $SS = 2072$, $F = 1.0$, $p = 0.33$). Similarly, while there was a trend towards lower seed set in flowers that received heterospecific pollen (flowers visited by moths that had previously visited heterospecific flowers) than flowers receiving only conspecific pollen (flowers visited by moths that had not previously visited heterospecific flowers), this difference was also not statistically significant (ANOVA: *D. wrightii*, $n = 13$ fruit, $SS = 1685$, $F = 0.55$, $p = 0.48$; *D. discolor*, $n = 22$ fruit, $SS = 247$, $F = 0.11$, $p = 0.74$). The two *Datura* species are not interfertile. Pollination benefits saturated rapidly with increasing floral visits for both *Datura* species: only a single moth visit is required for flowers of both *Datura* species to produce their maximum seed set. From a modeling perspective, these results simplify the models by precluding the need for complicated functions describing the probability of pollen transfer or heterospecific pollen interference.

For both *Datura* species, oviposition on each individual plant (the number of eggs that a female *M. sexta* laid) increased linearly with the number of floral visits that the moth made to that plant when moths made at least one floral visit (linear regression; *D. wrightii*, $n = 34$ plants, $t = 6.37$, $p < 0.001$; *D. discolor*, $n = 24$ plants, $t = 2.14$, $p = 0.04$). That is, for every *M. sexta* floral visit that a *Datura* plant receives, the number of eggs deposited on it increases linearly. Saturating functions failed to fit the data (nonlinear regression; *D. wrightii*, $n = 34$ plants, $t = 0$, $p = 1$; *D. discolor*, $n = 24$ plants, $t = 0.6$, $p = 0.57$). The slope of the linear regression gives the number of eggs that a female *M. sexta* deposits per floral visit (e_i in the model; see "Model parameterization" in the Methods of the main text), which does not differ significantly between *Datura* species (ANOVA; $n = 58$ plants, $SS = 7.1$, $F = 0.22$, $p = 0.64$). That is, given a floral visit, a moth is equally likely to oviposit and lay similar numbers of eggs on *D. wrightii* and *D. discolor*. In total, however, *M. sexta* lays significantly more eggs on *D. wrightii* than on *D. discolor*[1]. This oviposition bias towards *D. wrightii* is due to *M. sexta*'s preference for visiting *D. wrightii* flowers: *D. wrightii* receives 70% of *M. sexta* floral visits, but also 70% of *M. sexta* oviposition. From a modeling perspective, these results suggest that floral visitation is a good predictor of oviposition, thus precluding the need for complicated functions describing *M. sexta* foraging decisions for nectar sources versus larval host plants.

Supplementary Figure 1. Bifurcation diagrams show the equilibrium larval density of *M. sexta* on *D. wrightii* (L^*_w) and on *D. discolor* (L^*_d) with increasing defense ($h_{max,i} – h_i$). Red lines show the equilibrium larval density of the ancestral insect on *D. wrightii* (panel a) and *D. discolor* (panel b). The solid and dotted black lines give larval density in the pollination-herbivory system at the stable, high-density (coexistence) equilibrium and the unstable, low-density equilibrium associated with the Allee threshold, respectively. The point on the x-axis where the red and dotted black lines converge is the interaction transition boundary (Eq. 2 in the main text) and the point on the x-axis where the solid and dotted black lines converge is the interaction breakdown boundary (approximated by Eq. 3 in the main text). All other parameter values are given in Supplementary Tables 1,2 or were held at their final coESSs in Supplementary Table 3.

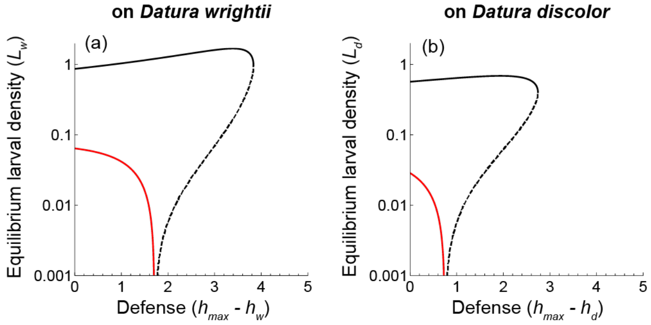

Supplementary Figure 2. Coevolutionary dynamics of plant and insect traits driving transitions from antagonism to mutualism in the one-plant species communities (Fig. 2e,f). Panels (a-c) are for *D. wrightii* and Panels (d-f) are for *D. discolor*. Panels (a,d) plot plant traits underlying pollination benefits from the antagonist, b_i (x_i^B; green lines), attraction (x_i^V; orange lines), and defense (x_i^H; gray lines) over evolutionary time, τ. Panels (b,e) plot insect traits underlying attraction (y_i^V; dashed orange lines) and defense (y_i^H; dashed gray lines). In the model, the coevolution of attraction (via x_i^V and y_i^V) affects visitation rate, v_i, while the coevolution of defense (via x_i^H and y_i^H) affects herbivory rate, h_i. Panels (c,f) plot the coevolutionary dynamics of pollination benefits (b_i; black lines), attraction (v_i; blue lines), and defense (h_i; purple lines) and are the same as Figure 2e,f in the main text, respectively.

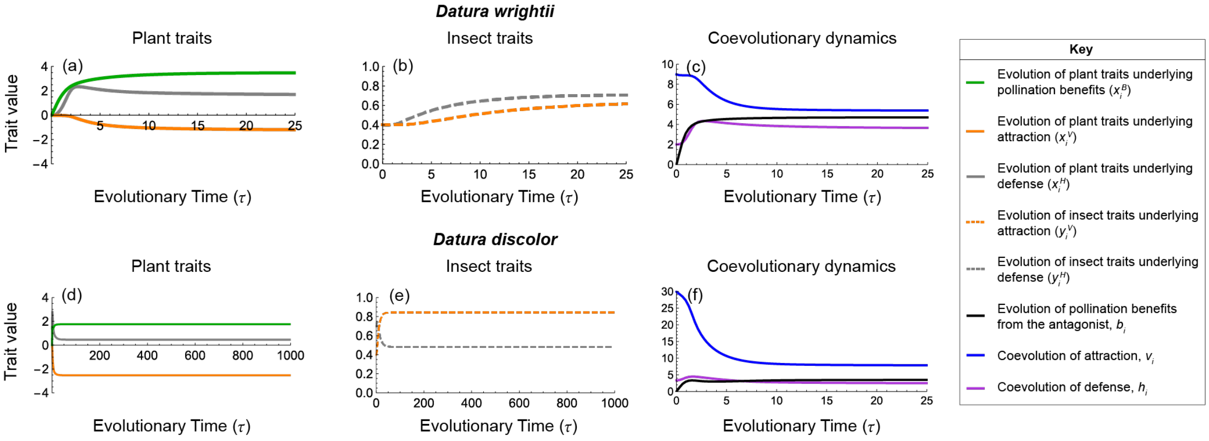

Supplementary Figure 3. Varying the costs associated with host and partner traits for different trade-off shapes reveals coevolutionary outcomes in the general model (Eq. 5 in the main text). Host costs associated with attraction ($c_{H,i}{}^{V}$) and defense ($c_{H,i}{}^{H}$) are varied in panels (a-c) and partner costs associated with visitation ($c_F{}^{V}$) and antagonism ($c_F{}^{H}$) are varied in panels (d-f). Panels (a,d) are for linear trade-offs (s = 1; subscripts and superscripts dropped for convenience), panels (b,e) are for convex trade-offs (s = 1.2), and panels (c,f) are for concave trade-offs (s = 0.95). Green regions indicate evolutionary transitions from antagonism to net mutualism. Gray regions indicate that net antagonism persists despite the co-option of the partner. White regions denote evolutionary purging of the partner. Black dots give the evolutionary parameter values used in the general model.

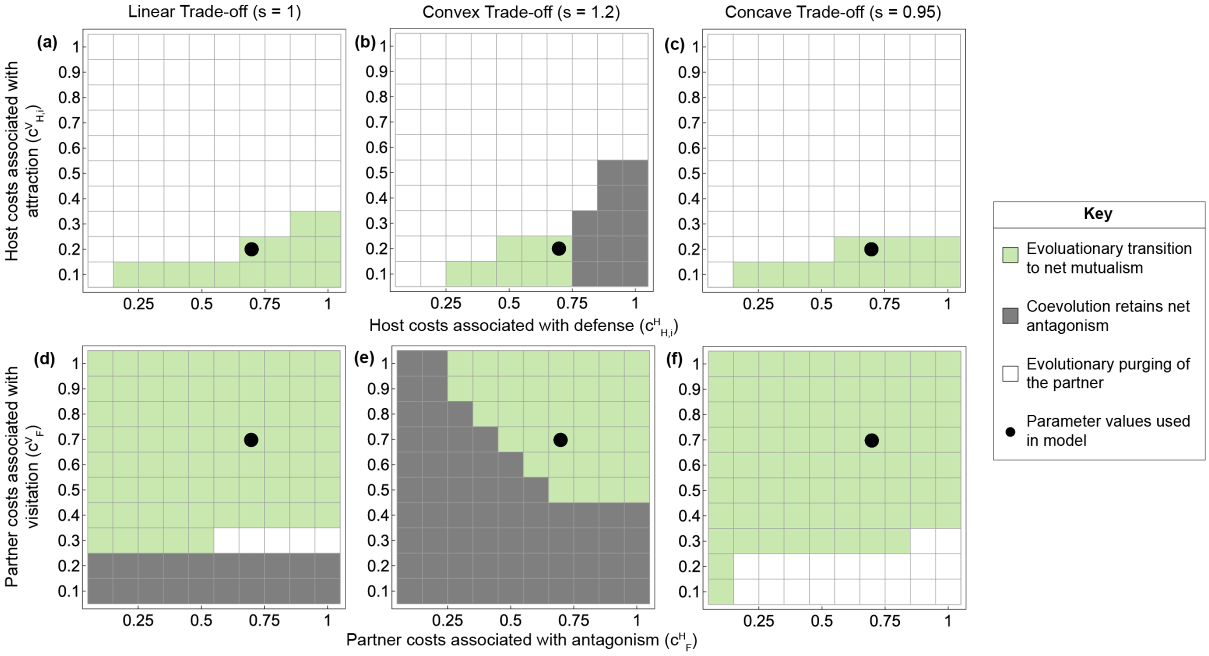

Supplementary Table 1. Parameters in the ecological model (Eq. 9). Parameters in bold coevolve in the model and values in bold are independent empirical estimates used to validate model predictions. (See "Model parameterization" in Methods of the main text for more information).

Symbol	Description	Value (*D. wrightii*)	Value (*D. discolor*)	Value (*P. parviflora*)
P_i	Plant density	State Variable		
L_i	Insect larval density per plant of species *i*	State Variable		
A	Insect adult density	State Variable		
$\mathbf{b_i}$	**Pollination benefits from *M. sexta***	**4.6 ± 0.2**	**3.6 ± 0.1**	–
$\mathbf{v_i}$	**Visitation rate**	**4.3 ± 0.6**	**2.3 ± 0.4**	0
$\mathbf{h_i}$	**Herbivory rate**	**1 ± 0.4**	**2 ± 0.8**	–
$\mathbf{e_i}$	**Oviposition efficiency**	**0.6 ± 0.1**	**0.6 ± 0.2**	–
H	Half-saturation constant	0.1		–
m_i	Maturation efficiency	0.005	0.003	0.01
d_i	Larval mortality rate	0.25	0.3	0.25
d_A	Adult mortality rate	0.2		
ε	Increased oviposition due to *A. palmeri*	3[a]		

[a] In the absence of *Agave palmeri*, $\varepsilon = 1$.

Supplementary Table 2. Evolutionary parameter values. Variables in bold evolve in the model.

Trait evolving	Parameter	Description	Value (*D. wrightii*)	Value (*D. discolor* and *P. parviflora*)
All Traits	τ	Evolutionary timescale	State variable	
	μ_x	Rate of plant evolution	1	
	μ_y	Rate of insect evolution	1	
	$W_{P,i}$	Invasion fitness of mutant plant	Equation 11a	
	$W_{I,i}$	Invasion fitness of mutant insect	Equation 11b	
Pollination benefits (b_i)	$\boldsymbol{x_i^B}$	**Underlying plant trait (e.g., flower size or shape)**	**Evolves**	
	$b_{max,i}$	Maximum value	5 [a,c]	
	B_i'	Saturation constant	1	
	$c_{P,i}^B$	Costliness of trait x_i^B	0.1 [a]	0.5 [a]
Attraction (v_i)	$\boldsymbol{x_i^V}$	**Underlying plant trait (e.g., production of floral volatiles)**	**Evolves**	
	$\boldsymbol{y_i^V}$	**Underlying insect trait (e.g., sensitivity to olfactory cues)**	**Evolves**	
	$v_{max,i}$	Maximum value	15 [b]	
	V_i'	Coefficient relating x_i^V and y_i^V to v_i	1	
	$c_{P,i}^V$	Costliness of trait x_i^V	0.25 [a]	0.4 [a,d]
Defense (h_i)	$\boldsymbol{x_i^H}$	**Underlying plant trait (e.g., production of chemical defenses)**	**Evolves**	
	$\boldsymbol{y_i^H}$	**Underlying insect trait (e.g., tolerance to plant secondary compounds)**	**Evolves**	
	$h_{max,i}$	Maximum value	5	
	H_i'	Coefficient relating x_i^H and y_i^H to h_i	1	
	$c_{P,i}^H$	Costliness of trait x_i^H	0.8	0.5
Oviposition efficiency (e_i)	$e_{max,i}$	Maximum value	1	
	$c_{I,i}^V$	Costliness of trait y_i^V	0.5	
	$c_{I,i}^H$	Costliness of trait y_i^H	0.5	

[a] Parameter values set to zero for the ancestral interaction.
[b] For *D. discolor*, $v_{max,i}$ = 50 in the one-plant species community and in the presence of *A. palmeri* because with lower values, *D. discolor* always evolutionarily purges *M. sexta*.
[c] For *P. parviflora*, $b_{max,i} = 0$ because *M. sexta* does not pollinate it
[d] For *P. parviflora*, $c_{P,i}^V = 0$ because there is no selection on *P. parviflora* for traits underlying floral visitation because *M. sexta* does not visit the flowers of *P. parviflora*.

Supplementary Table 3. Coevolutionary stable states (coESSs) predicted by the eco-coevolutionary models. Arrows (→) delineate between ancestral coESSs and the final coESSs.

Community	Fig.	Trait	*D. wrightii*				*D. discolor*			
			Trait (Ancestral)	Trait (Final)	coESS	Empirical Estimate	Trait (Ancestral)	Trait (Final)	coESS	Empirical Estimate
One *Datura* species	2a,b	v_i	x_w^V: 0<br>y_w^V: 0.4	x_w^V: −1.2<br>y_w^V: 0.6	9 → 5.4	–	x_d^V: 0<br>y_d^V: 0.4	x_d^V: −2.5<br>y_d^V: 0.8	30 → 7.8	–
		h_i	x_w^H: 0<br>y_w^H: 0.4	x_w^H: 1.7<br>y_w^H: 0.7	3 → 1.4	1 ± 0.4	x_d^H: 1.4<br>y_d^H: 0.7	x_d^H: 0.5<br>y_d^H: 0.5	1.6 → 2.5	2 ± 0.8
		b_i	x_w^B: 0	x_w^B: 3.5	0 → 4.7	4.6 ± 0.2	x_d^B: 0	x_d^B: 1.8	0 → 3.5	3.6 ± 0.1
		e_i	–	–	0.9 → 0.6	0.6 ± 0.1	–	–	0.7 → 0.6	0.6 ± 0.2
			D. wrightii (in the presence of *D. discolor*)				*D. discolor* (in the presence of *D. wrightii*)			
Both *Datura species*	3a,b	v_i	x_w^V: 0<br>y_w^V: 0.4	x_w^V: −1.6<br>y_w^V: 0.7	9 → 4.3	4.3 ± 0.6	x_d^V: 0<br>y_d^V: 0.4	x_d^V: −2.5<br>y_d^V: 0.8	9 → 2.3	2.3 ± 0.4
		h_i	x_w^H: 0<br>y_w^H: 0.4	x_w^H: 1.8<br>y_w^H: 0.7	3 → 1.3	1 ± 0.4	x_d^H: 1.6<br>y_d^H: 0.7	x_d^H: 0.5<br>y_d^H: 0.5	1.4 → 2.5	2 ± 0.8
		b_i	x_w^B: 0	x_w^B: 3.5	0 → 4.7	4.6 ± 0.2	x_d^B: 0	x_d^B: 1.8	0 → 3.5	3.6 ± 0.1
		e_i	–	–	0.9 → 0.6	0.6 ± 0.1	–	–	0.7 → 0.6	0.6 ± 0.2
			D. wrightii				*P. parviflora*			
D. wrightii + *P. parviflora*	4a	v_i	x_w^V: 0<br>y_w^V: 0.4	x_w^V: −1.5<br>y_w^V: 0.7	9 → 4.6	–	x_p^V: 0<br>y_p^V: 0.4	x_p^V: 0<br>y_p^V: 0.4	9 → 9	–
		h_i	x_w^H: 1.7<br>y_w^H: 0.7	x_w^H: 1.8<br>y_w^H: 0.7	1.4 → 1.3	1 ± 0.4	x_p^H: 2.5<br>y_p^H: 0.8	x_p^H: 3.6<br>y_p^H: 0.9	0.8 → 0.3	–
		b_i	x_w^B: 0	x_w^B: 3.5	0 → 4.7	4.6 ± 0.2	–	–	0	–
		e_i	–	–	0.7 → 0.6	0.6 ± 0.1	–	–	0.7 → 0.6	–
			P. parviflora				*D. discolor*			
D. discolor + *P. parviflora*	4b	v_i	x_p^V: 0<br>y_p^V: 0.4	x_p^V: 0<br>y_p^V: 0.4	9 → 9	–	x_d^V: 0<br>y_d^V: 0.4	x_d^V: −1.7<br>y_d^V: 0.7	9 → 4	–
		h_i	x_p^H: 2.1<br>y_p^H: 0.8	x_p^H: 2.9<br>y_p^H: 0.9	1 → 0.6	–	x_d^H: 1.8<br>y_d^H: 0.7	x_d^H: 0.1<br>y_d^H: 0.4	1.3 → 2.9	2 ± 0.8
		b_i	–	–	0	–	x_d^B: 0	x_d^B: 1.8	0 → 3.6	3.6 ± 0.1
		e_i	–	–	0.7 → 0.6	–	–	–	0.7 → 0.7	0.6 ± 0.2
			D. wrightii (in the presence of *A. palmeri*)				*D. discolor* (in the presence of *A. palmeri*)			
Each *Datura* species + *A. palmeri*	4c,d	v_i	x_w^V: 0<br>y_w^V: 0.4	x_w^V: −1.2<br>y_w^V: 0.6	9 → 5.3	–	x_d^V: 0<br>y_d^V: 0.4	x_d^V: −2.6<br>y_d^V: 0.8	30 → 7.5	–
		h_i	x_w^H: 2<br>y_w^H: 0.8	x_w^H: 3.1<br>y_w^H: 0.9	1.1 → 0.5	1 ± 0.4	x_d^H: 3<br>y_d^H: 0.9	x_d^H: 2.1<br>y_d^H: 0.8	0.6 → 1.1	2 ± 0.8
		b_i	x_w^B: 0	x_w^B: 3.4	0 → 4.7	4.6 ± 0.2	x_d^B: 0	x_d^B: 1.5	0 → 3.1	3.6 ± 0.1
		e_i	–	–	0.7 → 0.5	0.6 ± 0.1	–	–	0.6 → 0.5	0.6 ± 0.2

Supplementary References

1. Smith, G. P., Johnson, C. A., Davidowitz, G. & Bronstein, J. L. Linkages between nectaring and oviposition preferences of *Manduca sexta* on two co-blooming *Datura* species in the Sonoran Desert. *Ecol. Entomol.* **43**, 85–92 (2018).
2. Bronstein, J. L., Huxman, T., Horvath, B., Farabee, M. & Davidowitz, G. Reproductive biology of *Datura wrightii*: The benefits of a herbivorous pollinator. *Ann. Bot.* **103**, 1435–1443 (2009).
