# Supplementary material for: Coevolutionary transitions from antagonism to mutualism explained by the Co-Opted Antagonist Hypothesis
Source: Nat Commun. 2021 May 17;12:2867. doi: 10.1038/s41467-021-23177-x (PMC8129128; doi:10.1038/s41467-021-23177-x)
Supplement: Supplementary file 3 — Reporting Summary [file 41467_2021_23177_MOESM3_ESM.pdf]

nature research

Corresponding author(s): Christopher Johnson

Last updated by author(s): Mar 22, 2021

Reporting Summary

Nature Research wishes to improve the reproducibility of the work that we publish. This form provides structure for consistency and transparency in reporting. For further information on Nature Research policies, see our Editorial Policies and the Editorial Policy Checklist.

Statistics

For all statistical analyses, confirm that the following items are present in the figure legend, table legend, main text, or Methods section.

n/a	Confirmed	
☐	☒	The exact sample size (*n*) for each experimental group/condition, given as a discrete number and unit of measurement
☒	☐	A statement on whether measurements were taken from distinct samples or whether the same sample was measured repeatedly
☐	☒	The statistical test(s) used AND whether they are one- or two-sided<br>*Only common tests should be described solely by name; describe more complex techniques in the Methods section.*
☒	☐	A description of all covariates tested
☒	☐	A description of any assumptions or corrections, such as tests of normality and adjustment for multiple comparisons
☐	☒	A full description of the statistical parameters including central tendency (e.g. means) or other basic estimates (e.g. regression coefficient) AND variation (e.g. standard deviation) or associated estimates of uncertainty (e.g. confidence intervals)
☐	☒	For null hypothesis testing, the test statistic (e.g. F, t, r) with confidence intervals, effect sizes, degrees of freedom and P value noted<br>*Give P values as exact values whenever suitable.*
☒	☐	For Bayesian analysis, information on the choice of priors and Markov chain Monte Carlo settings
☒	☐	For hierarchical and complex designs, identification of the appropriate level for tests and full reporting of outcomes
☒	☐	Estimates of effect sizes (e.g. Cohen's *d*, Pearson's *r*), indicating how they were calculated

Our web collection on statistics for biologists contains articles on many of the points above.

Software and code

Policy information about availability of computer code

Data collection	Microsoft Excel (v. 16.48)
Data analysis	R (v. 3.1.0) and Mathematica (v. 12.0). Mathematica codes are available on Zenodo: https://doi.org/10.5281/zenodo.4628187

For manuscripts utilizing custom algorithms or software that are central to the research but not yet described in published literature, software must be made available to editors and reviewers. We strongly encourage code deposition in a community repository (e.g. GitHub). See the Nature Research guidelines for submitting code & software for further information.

Data

Policy information about availability of data

All manuscripts must include a data availability statement. This statement should provide the following information, where applicable:

- Accession codes, unique identifiers, or web links for publicly available datasets
- A list of figures that have associated raw data
- A description of any restrictions on data availability

Data used to parameterize and validate the models are available within the paper and its Supplementary Information as well as on Zenodo: https://doi.org/10.5281/zenodo.4628187. Our data was recorded in Microsoft Excel (v. 16.48) and was analyzed in R (v. 3.1.0).

Field-specific reporting

Please select the one below that is the best fit for your research. If you are not sure, read the appropriate sections before making your selection.

☐ Life sciences ☐ Behavioural & social sciences ☒ Ecological, evolutionary & environmental sciences

For a reference copy of the document with all sections, see nature.com/documents/nr-reporting-summary-flat.pdf

Ecological, evolutionary & environmental sciences study design

All studies must disclose on these points even when the disclosure is negative.

Study description	Data were used to parameterize or validate a mathematical model. Most data were obtained from the literature as discussed in the Methods. Some of these data came from our experiment, which is summarized in Supplementary Data 1 and described in Smith et al. 2018 (https://doi.org/10.1111/een.12475) as well as in the boxes below.
Research sample	We performed preference assays to evaluate Manduca sexta floral visitation and oviposition preferences. M. sexta individuals (female and male) from the University of Arizona laboratory colony were sequentially released into a large (22m x 7.5m x 3m) greenhouse containing two potted D. wrightii and two potted D. discolor plants arranged in a checkered array with ~2m between plants.
Sampling strategy	Sample size was determined by the number of adult Manduca sexta that made at least one floral visit or oviposit. A total of 135 moths were included.
Data collection	Data were collected by Christopher A Johnson and Gordon P. Smith.
Timing and spatial scale	The experiment was performed between July–September, 2014, and August–September, 2015. The greenhouse in which the experiment was conducted was 22m x 7.5m x 3m.
Data exclusions	Data were excluded in cases in which the moth did not nectar or oviposit on any plant.
Reproducibility	The experiment was performed over multiple nights within a season as well as over two summers (2014 and 2015). Moths exhibited similar behaviors across these multiple trials, verifying the reproducibility of the experimental methods.
Randomization	The diagonal positions of the different plant species in the checkered array within the experimental chamber were randomized each night. The order in which the moths were released each night was also randomized.
Blinding	The investigators were not blind because there were no experimental groups per se in the experiment.

Did the study involve field work? ☐ Yes ☒ No

Reporting for specific materials, systems and methods

We require information from authors about some types of materials, experimental systems and methods used in many studies. Here, indicate whether each material, system or method listed is relevant to your study. If you are not sure if a list item applies to your research, read the appropriate section before selecting a response.

Materials & experimental systems

n/a	Involved in the study	
☒	☐	Antibodies
☒	☐	Eukaryotic cell lines
☒	☐	Palaeontology and archaeology
☐	☒	Animals and other organisms
☒	☐	Human research participants
☒	☐	Clinical data
☒	☐	Dual use research of concern

Methods

n/a	Involved in the study	
☒	☐	ChIP-seq
☒	☐	Flow cytometry
☒	☐	MRI-based neuroimaging

Animals and other organisms

Policy information about studies involving animals; ARRIVE guidelines recommended for reporting animal research

Laboratory animals	M. sexta hawkmoth individuals (female and male; ages 2-5 days) were used from the University of Arizona laboratory colony.
Wild animals	N/A

Field-collected samples	N/A
Ethics oversight	No ethical approval was necessary because the study organism is an insect; however, researchers (C.A.J. and G.P.S.) received training in rearing and handling moths from staff at the University of Arizona laboratory colony.

Note that full information on the approval of the study protocol must also be provided in the manuscript.
