# Supplementary material for: Coevolutionary transitions from antagonism to mutualism explained by the Co-Opted Antagonist Hypothesis
Source: Nat Commun. 2021 May 17;12:2867. doi: 10.1038/s41467-021-23177-x (PMC8129128; doi:10.1038/s41467-021-23177-x)
Supplement: Supplementary file 4 — Description of Additional Supplementary Files [file 41467_2021_23177_MOESM4_ESM.pdf]

Description of Additional Supplementary Files

File Name: Supplementary Movie 1
Description: **Coevolutionary dynamics of *Manduca sexta* and *Datura wrightii* in a one-plant species community (corresponding with Figure 2a in the main text).** The movie shows the expansion of the green mutualistic region with the evolution of pollination benefits and simultaneous coevolution of attraction and defense to their coESSs over evolutionary time, τ. For illustrative purposes, movie frames 1-16 show coevolution from τ = 0 to τ = 3, frames 16-20 show coevolution from τ = 3 to τ = 5, frames 21-25 show coevolution from τ = 5 to τ = 10, and frames 26-30 show coevolution from τ = 10 to τ = 25.

File Name: Supplementary Movie 2
Description: **Coevolutionary dynamics of *Manduca sexta* and *Datura discolor* in a one-plant species community (corresponding with Figure 2b in the main text).** The movie shows the expansion of the green mutualistic region with the evolution of pollination benefits and simultaneous coevolution of attraction and defense to their coESSs over evolutionary time, τ. For illustrative purposes, movie frames 1-11 show coevolution from τ = 0 to τ = 2, frames 12-19 show coevolution from τ = 2 to τ = 10, and frames 20-25 show coevolution from τ = 10 to τ = 25.

File Name: Supplementary Movie 3
Description: **Coevolutionary dynamics of *Manduca sexta* and both *Datura* species in the two-plant species community (corresponding with Figures 3a,b in the main text).** The movie shows the expansion of the green mutualistic region with the evolution of pollination benefits and simultaneous coevolution of attraction and defense to their coESSs over evolutionary time, τ. For illustrative purposes, movie frames 1-11 show coevolution from τ = 0 to τ = 2, frames 12-19 show coevolution from τ = 2 to τ = 10, and frames 20-24 show coevolution from τ = 10 to τ = 50.

File Name: Supplementary Movie 4
Description: **Coevolutionary dynamics of *Manduca sexta*, each *Datura* species, and the alternative larval host plant (corresponding with Figures 4a,b in the main text).** The movie shows the expansion of the green mutualistic region with the evolution of pollination benefits and simultaneous coevolution of attraction and defense to their coESSs over evolutionary time, τ. While the movies run simultaneously, panels (a) and (b) show separate models in which each *Datura* species coevolves with the alternative larval host plant and not with the other *Datura* species. For illustrative purposes, movie frames 1-11 show coevolution from τ = 0 to τ = 2, frames 12-19 show coevolution from τ = 2 to τ = 10, and frames 20-24 show coevolution from τ = 10 to τ = 50.

File Name: Supplementary Movie 5
Description: **Coevolutionary dynamics of *Manduca sexta*, each *Datura* species, and the alternative nectar source (corresponding with Figures 4c,d in the main text).** The movie shows the expansion of the green mutualistic region with the evolution of pollination benefits and simultaneous coevolution of attraction and defense to their coESSs over evolutionary time, τ. While the movies run simultaneously, panels (a) and (b) show separate models in which each *Datura* species coevolves in the presence of the alternative nectar

source and not with the other *Datura* species. For illustrative purposes, movie frames 1-11 show coevolution from τ = 0 to τ = 2, frames 12-19 show coevolution from τ = 2 to τ = 10, and frames 20-25 show coevolution from τ = 10 to τ = 25.

File Name: Supplementary Data 1
Description: **Data from our experiments on floral visitation and oviposition of *Manduca sexta* hawkmoths at *Datura wrightii* and *Datura discolor*.** The first dataset (“Seed Set” tab) gives the number of floral visits that each plant received from *M. sexta*, its seed set, whether it was visited by at least one moth (“Moth-Pollinated”), whether it was potentially cross-pollinated (“Cross-Pollinated”), and whether it potentially received heterospecific pollen (“Heterospecific Pollen”). The second dataset (“Floral Visits” tab) gives the total number of floral visits that each plant received from all moths. The third dataset (“Oviposition vs visitation” tab) gives the number of floral visits and number of eggs oviposited on each plant that received at least one floral visit.
